# The Tumour Glyco‐Code: Sialylation as a Mediator of Stromal Cell Immunosuppression in the Tumour Microenvironment

**DOI:** 10.1002/eji.70000

**Published:** 2025-07-16

**Authors:** Aoise O'Neill, Norashikin Zakaria, Hannah Egan, Oliver Treacy, Aisling M. Hogan, Michael O'Dwyer, Sean O. Hynes, Aideen E. Ryan

**Affiliations:** ^1^ Discipline of Pharmacology and Therapeutics, School of Medicine, College of Medicine, Nursing and Health Sciences University of Galway Galway Ireland; ^2^ Regenerative Medicine Institute (REMEDI), School of Medicine, College of Medicine Nursing and Health Sciences University of Galway Galway Ireland; ^3^ Lambe Institute for Translational Research, School of Medicine, College of Medicine, Nursing and Health Sciences University of Galway Galway Ireland; ^4^ Department of Colorectal Surgery Galway University Hospital Galway Ireland; ^5^ Discipline of Medicine, School of Medicine, College of Medicine, Nursing and Health Sciences University of Galway Ireland; ^6^ Division of Anatomical Pathology Galway University Hospital Galway Ireland; ^7^ Discipline of Pathology, School of Medicine, College of Medicine, Nursing and Health Sciences University of Galway Galway Ireland; ^8^ CÚRAM Centre for Research in Medical Devices, School of Medicine, College of Medicine, Nursing and Health Sciences University of Galway Galway Ireland

**Keywords:** cancer‐associated fibroblasts, immunosuppression, sialylation, stromal cells, tumour microenvironment

## Abstract

The tumour microenvironment (TME) comprises a complex interplay of tumour cells, nonmalignant cells (including endothelial, immune, and stromal cells), and secreted factors within the extracellular matrix (ECM). Immunosuppression within the TME significantly hinders the efficacy of cancer immunotherapies. Stromal‐rich TMEs, characterised by an abundance of mesenchymal stromal cells (MSCs) and cancer‐associated fibroblasts (CAFs), are particularly immunosuppressive and associated with poor responses to conventional and immune‐based therapies. Glycans, carbohydrate structures on cell surfaces, are dynamically regulated during tumourigenesis and mediate crucial cell–cell communications through receptor–ligand interactions. Sialylation, the addition of sialic acids to glycans, forms sialoglycans that can engage inhibitory Siglec receptors expressed on immune cells and promote immunosuppressive signalling. Emerging evidence implicates aberrant sialylation in the TME as a key driver of immunosuppression. More recently, sialylation of stromal cells in the TME has been shown to suppress anti‐tumor immunity. This review explores the role of sialylation within stromal‐rich, immunosuppressive TMEs, focusing on how specific sialic acid/Siglec interactions dictate innate and adaptive immune responses. We discuss the potential of targeting glycoimmune checkpoints to overcome stromal‐mediated resistance and enhance anti‐tumour immunity.

## Graphical abstract

The Sialic acid/Siglec axis is emerging as an important target in the TME. We explore the role of sialylation within stromal‐rich, immunosuppressive TMEs, focusing on how specific sialic acid/Siglec interactions dictate innate and adaptive immune responses. We propose that targeting glycoimmune checkpoints as a mechanism may overcome stromal‐mediated therapy resistance and enhance anti‐tumour immunity.

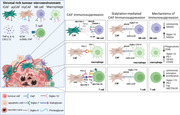

## 1 Introduction

Immunotherapeutics have revolutionised the treatment of cancer in recent years. A major barrier to their efficacy are immunosuppressive TMEs. The cancer‐immunity cycle has been updated to reflect the complexities of the TME, including the effects of stroma and sialylation on anti‐tumour immune response [1]. The stromal compartment of the TME is highly heterogeneous, consisting of non‐malignant, non‐haematopoietic cells including mesenchymal stromal cells (MSCs), cancer‐associated fibroblasts (CAFs), endothelial cells, pericytes and adipocytes [2, 3, 4]. In stromal‐rich tumours, MSCs and CAFs mediate ECM deposition, therapeutic resistance and immunosuppression [5, 6, 7, 8, 9]. Stromal signatures are associated with poor response to chemotherapy and low relapse‐free survival rates in many cancers [10, 11, 12]. Understanding the mechanisms of stromal‐mediated immunosuppression is crucial to the development of innovative strategies to increase the efficacy of immunotherapies in stromal‐rich tumours, such as colorectal cancer, pancreatic, lung and ovarian cancer.

The cancer “glyco‐code” refers to aberrant post‐translational modifications (PTMs) that instruct core biological processes and progression in cancer [13]. Aberrant glycosylation in the TME is associated with many of the hallmarks of cancer, including resistance to therapy and immune evasion [14, 15]. Sialylation is a process where sialic acids are attached to glycan chains via glycosidic bonds, creating sialoglycans on glycoproteins and gangliosides on glycolipids [16, 17, 18]. These glycans play both a structural role (glycocalyx) and an informational role (glyco‐code) in cancer [19]. Sialoglycans, through their negative charge, regulate cell–cell interactions, ECM interactions, drug resistance and immunosuppression in the TME. However, knowledge on the biological consequences of altered sialylation in the TME is limited [20]. Here, we review sialylation as a mediator of stromal‐immune interactions and a novel target of immunosuppression in the TME.

PTMs of proteins and lipids add another layer of complexity to TME interactions and can dramatically alter cellular function. Aberrant glycosylation, the enzymatic addition of carbohydrate structures (glycans) to biomolecules, represents a hallmark of cancer, often referred to as the cancer “glyco‐code” [13]. These altered glycan structures on cancer cells and within the TME are deeply implicated in core biological processes underpinning cancer progression, including sustained proliferation, invasion, metastasis, angiogenesis, resistance to therapy, and, importantly, immune evasion [14, 15]. There is also an increasing appreciation for the potential impact of PTMs in immune checkpoint signalling in the TME [21, 22].

## 2 Sialylation: The Tumour Glyco‐Code

Sialic acids are negatively charged nine‐carbon sugars, typically added to glycoproteins and glycolipids (forming sialoglycans and gangliosides, respectively) by enzymes called sialyltransferases. On the other hand, neuraminidases cleave or remove these sialic acids, and the balance of activity of sialyltransferases and neuraminidases determines the overall level of sialic acid [23]. The resulting dense layer of sialylated molecules on the cell surface, part of the glycocalyx, plays crucial roles in mediating cell‐cell and cell‐ECM interactions [19]. Beyond structural roles, sialoglycans act as key signalling molecules, regulating cellular communication and immune responses. Specifically, sialic acids can serve as ligands for Siglecs (sialic acid‐binding immunoglobulin‐type lectins), a family of receptors expressed predominantly on immune cells. Engagement of inhibitory Siglecs by sialoglycans typically dampens immune cell activation, representing a critical mechanism of immune regulation and self‐tolerance that can be hijacked by cancer cells. Hypersialylation is frequently observed in cancer and is associated with immunologically “cold” tumours, characterised by poor immune infiltration, resistance to anti‐cancer therapies, and disease progression [24, 25, 26, 27].

Emerging studies have highlighted sialylation as a mediator of stromal cell immunosuppression in the TME [28, 29], although this area remains largely unexplored compared to our understanding of the impact of cancer cell sialylation on immune evasion. In this review, we highlight emerging evidence and contextualise sialylation as a mediator of stromal‐induced immunosuppression in the TME. We explore sialic acid modifications in tumour stromal components, focusing on CAFs and the modulation of immune cell function via the Siglec/sialic acid axis. Signalling through the Siglec/sialic acid axis can facilitate immune escape and hinder effective anti‐tumour responses. Understanding sialylation‐dependent stromal‐immune interactions may unveil novel therapeutic avenues targeting the glyco‐immune checkpoints within the TME.

## 3 Sialylation as a Hallmark of Cancer

**FIGURE 1:**
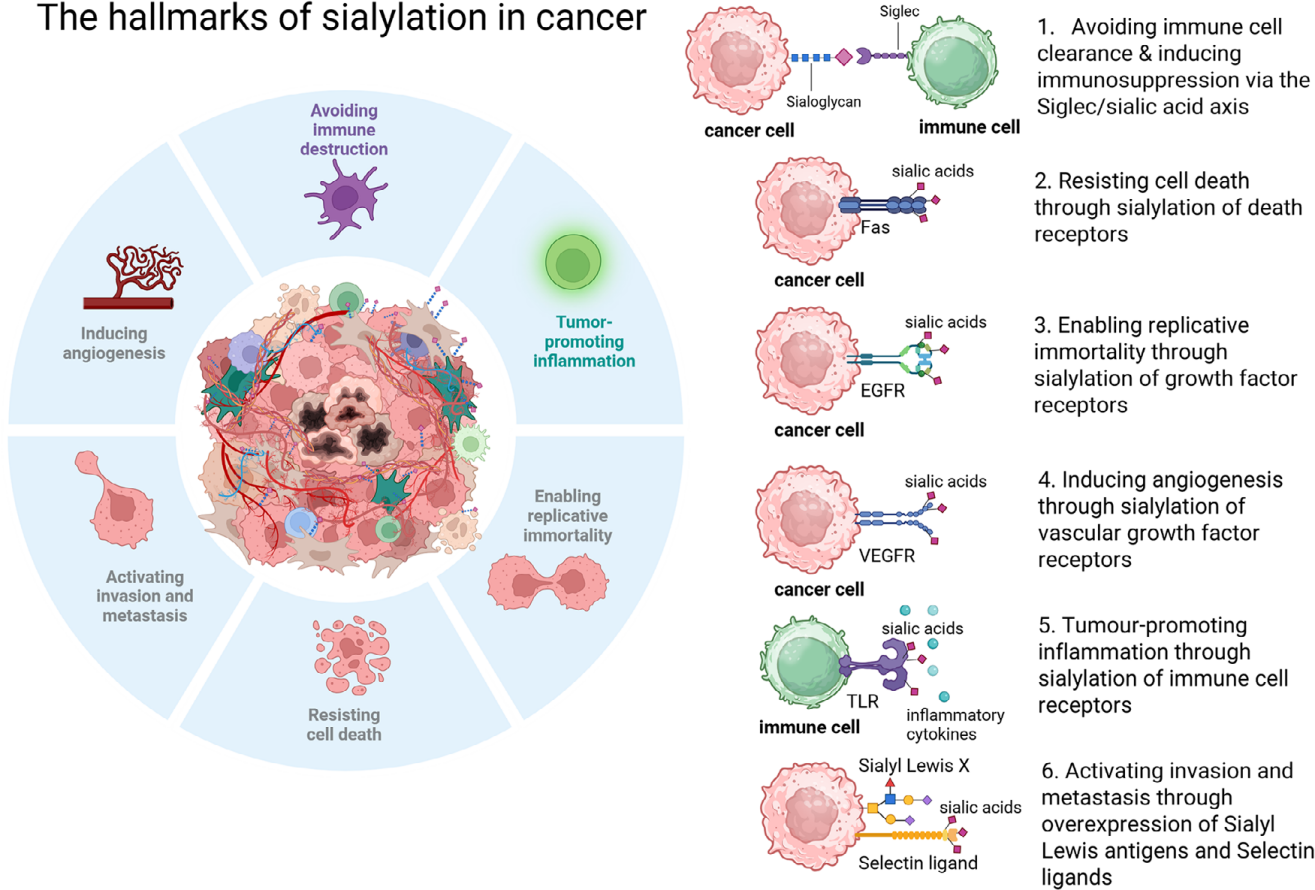
Sialylation and the hallmarks of cancer in the tumour microenvironment. Hanahan and Weinberg's Hallmarks of Cancer provides a framework overview of the characteristics of human tumours [32, 104]. Emerging evidence indicates that sialylation is associated with many of the hallmarks of cancer that promote tumorigenesis in the complex tumour microenvironment, including avoiding immune destruction, tumour‐promoting inflammation, enabling replicative immortality, resisting cell death, activating invasion and metastasis and inducing angiogenesis.

There is a growing body of evidence highlighting the functional impact of aberrant glycosylation in cancer [30, 31]; however, the specific contribution of sialylation to the individual hallmarks of cancer is only beginning to be understood. In Figure 1, we summarise and contextualise current evidence that sialylation impacts multiple hallmarks of cancer [32], including avoiding immune destruction, tumour‐promoting inflammation, enabling replicative immortality, resisting cell death, activating invasion and metastasis and inducing angiogenesis.

Cancer cells have a dense glycan shield (glycocalyx) rich in sialic acids that shields tumour cells from immune recognition by cytotoxic CD8+ T cells and natural killer (NK) cells, aiding the escape of cancer cells from immune recognition and clearance [33]. Sialylation plays a role in resisting cell death through sialylation of apoptosis‐regulating proteins, such as integrins, to enhance cancer cell survival [34]. Sialylation of death receptors such as Fas, mediated by sialyltransferase ST6Gal1, can inhibit Fas‐mediated apoptosis, promoting cell death resistance [35]. Sialylation impacts replicative immortality, playing a crucial role in maintaining stem‐like properties integral to replicative immortality in cancer stem cells (CSCs). CSC markers include the glycoprotein CD44 which, when bound, promotes cell proliferation and resistance to cellular senescence. Interestingly, CD44 has been recently identified as a ligand for Siglec‐15 [36]. Vascular endothelial growth factor receptor (VEGFR), a key player in the promotion of angiogenesis, has been shown to be hyperactivated when hypersialylated [37]. Sialylation shapes tumour‐promoting inflammation through sialylation of immune receptors such as toll‐like receptors (TLRs), which dampens their capacity for inflammatory signalling pathways [38]. Tumours, including CRC, have been shown to overexpress Sialyl–Lewis antigens, which are key drivers of metastasis, through enhanced endothelial cell interactions during vascular extravasation and detachment of primary tumour cells. Increased tumour–endothelial cell adhesion through selectin ligand binding enhanced migration and invasion [39, 40, 41, 42, 43, 44]. These known sialic acid‐dependent functional mechanisms highlight the fact that hypersialylation is an increasingly important target in the TME and may impact multiple mechanisms of tumour development and progression.

## 4 The Siglec/Sialic Acid Axis: An Immune Checkpoint

Sialylation plays an important role in determining self‐ versus non‐self‐entities in the body and tightly regulates immune activation through Siglec receptor engagement [45]. Siglec receptors are expressed on a wide range of immune cells. Human and mouse Siglecs may be divided into conserved and CD33‐related Siglecs. There are fourteen active human Siglecs expressed on immune cells, the majority of which are inhibitory receptors [38]. Conserved Siglecs include Siglec‐1, ‐2, ‐4 and ‐15, while human CD33‐related Siglecs include Siglec‐3, ‐5, ‐6, ‐7, ‐8, ‐9, ‐10, ‐11, ‐14 and ‐16. Mouse CD33‐related Siglecs include Siglec‐3 and the orthologues Siglec ‐E, ‐F, ‐G and ‐H. Inhibitory Siglec receptors possess an intracellular immunoreceptor tyrosine‐based inhibitory motif (ITIM) domain. [38]. Upon sialoglycan ligand binding, ITIMs recruit SHP‐1/‐2 phosphatases, which are responsible for downstream phosphorylation of key signalling intermediates involved in immune cell activation, dampening cell signalling pathways such as NF‐ĸB, ultimately reducing immune cell cytotoxicity, cytokine release and immune synapse formation [45]. This mechanism mediates T and B cell receptor signalling, NK cell activation and macrophage inflammatory signalling, resulting overall in a downregulation of immune activation [16, 46]. The process by which sialic acids are generated and regulated in cells and the role of sialic acids in human health and disease have been recently described in detail by Zhu et al. [47]. The Siglec/sialic acid interaction is exploited by cancer cells that exhibit elevated levels of sialylation. Tumour sialylation correlates with distinct immune cell populations (e.g. regulatory T cells (Tregs) and tumour‐associated macrophages (TAMs)), immune cell states (suppressed antigen presentation and T cell responses) and reduced survival in human cancers [48].

The engagement of inhibitory Siglecs by tumour‐associated sialoglycans can suppress immune cell effector functions, such as the cytotoxic activity of NK cells [49] and CD8+ T cells, inhibit phagocytosis by macrophages, impair antigen presentation by dendritic cells [50, 51], and modulate B cell responses. The sialic acid/Siglec axis functions analogously to well‐established immune checkpoints like the PD‐1/PD‐L1 axis to promote tumour immune escape [7, 52, 53]. Targeting this axis, either by blocking Siglec receptors or removing sialic acid ligands, is therefore emerging as a promising strategy in cancer immunotherapy to restore anti‐tumour immunity. AL009 (Alector), a Siglec‐7 antagonist monoclonal antibody, is in the early (pre‐clinical/phase 1) stage of clinical development for treating advanced solid tumours. E‐602 (Palleon Pharmaceuticals), a sialidase‐based enzyme therapy to remove sialic acids from tumour cells, is in phase 1/2 clinical trials (NCT05061590) to treat advanced solid tumours, including breast and pancreatic cancers, both alone and in combination with a PD‐1 mAb. As these therapies progress through clinical trials, they have the potential to become key targets to enhance immune infiltration and activation in immune‐excluded, therapy‐resistant tumours. Sialic acid impacts many tumour characteristics, with perhaps the most important being immune regulation [15, 54]. Hypersialylation of tumour antigens has been shown to regulate NK cell function in a Siglec‐7‐dependent manner in both breast cancer (BC) and multiple myeloma (MM) [20, 49]. It has been demonstrated that sialoglycans in the BC TME recruit NK cell inhibitory Siglec receptors to the immune synapse and reduce NK cell activation via the NKG2D receptor in a BC model [20]. Targeting sialoglycans on HER2+ breast cancer cells abolished Siglec ligands, increased activating NKG2D interactions and yielded increased NK cell cytotoxicity [20].

Targeting sialylation in cancer models has shown therapeutic efficacy and improved anti‐tumour immune responses. A study by Stanczak et al. [48] showed that targeted removal of Siglec ligands in the tumour microenvironment, using an antibody‐sialidase conjugate, enhanced adaptive antitumor immunity and halted tumour progression in several murine models. They identified Siglec‐E signalling on TAMs as one of the mechanisms of immunosuppression, and when targeted, they enhanced the efficacy of immune checkpoint blockade. In another study, targeting sialic acid using a sialyltransferase inhibitor in of breast cancer cells increased antibody‐dependent cellular phagocytosis (ADCP) by macrophages [50]. Cao et al. [55] showed in an in vitro model of ovarian cancer that knockdown of ST3Gal3 inhibited metastasis and repolarised TAMs from an anti‐inflammatory to a pro‐inflammatory, anti‐tumour phenotype. Many recent studies have highlighted the role of Siglec‐10 in immune modulation in cancer; however, the crystal structure of Siglec‐10 has not yet been experimentally resolved. This highlights the potential for novel glyco‐immune checkpoint discovery as this information becomes available.

A study from Lv et al. [51] uncovered Siglec‐10 as a driver of macrophage‐mediated immunosuppression in gastric cancer (GC). Targeting Siglec‐10+ macrophages in GC through Siglec‐10 blocking enhanced anti‐tumour immunity and synergistically improved response to anti‐PD‐1 immunotherapy in ex vivo tumour models. Wieboldt et al. [56] showed that overexpression of sialoglycans led to myeloid‐derived suppressor cells (MDSCs) overexpression of Siglec‐9 in lung cancer, which was directly linked to the secretion of CCL2, which in turn contributed to T cell suppression. Dual‐targeting of mouse Siglec‐E and PD‐L1 in vivo sustained anti‐tumour immune response and prevented tumour progression [48]. Combination therapies targeting the Siglec/sialic acid axis and actively recruiting cytotoxic immune cells may be the key to improving outcomes in poor response tumours. Sialylation of the Fc domain of IgG antibodies has been shown to impair complement‐dependent cytotoxicity (CDC), limiting pro‐inflammatory IgG effector functions [57]. Similarly, targeting ST3Gal1‐mediated upregulation of CD55 sialylation was shown to increase CDC of breast cancer cells and enhance sensitivity to ADCC [58]. These studies confirm that upregulation of sialoglycans in the TME represents a promising target for novel dual‐targeting immunotherapeutic approaches to enhance anti‐cancer immunity across different cancer types. Further knowledge on the effects of inflammation, hypoxia and metabolic changes in the TME are needed to design and tailor effective therapeutic strategies targeting the sialic acid/Siglec axis.

Sialylation has been recently identified as a driver of immunosuppression in stromal‐rich cancer. We review evidence for hypersialylation in stromal‐rich cancers and propose strategies for targeting stromal cell sialylation to overcome immunosuppression and therapeutic resistance.

## 5 Sialylation of CAFs: A Key Driver of Immunosuppression

**FIGURE 2:**
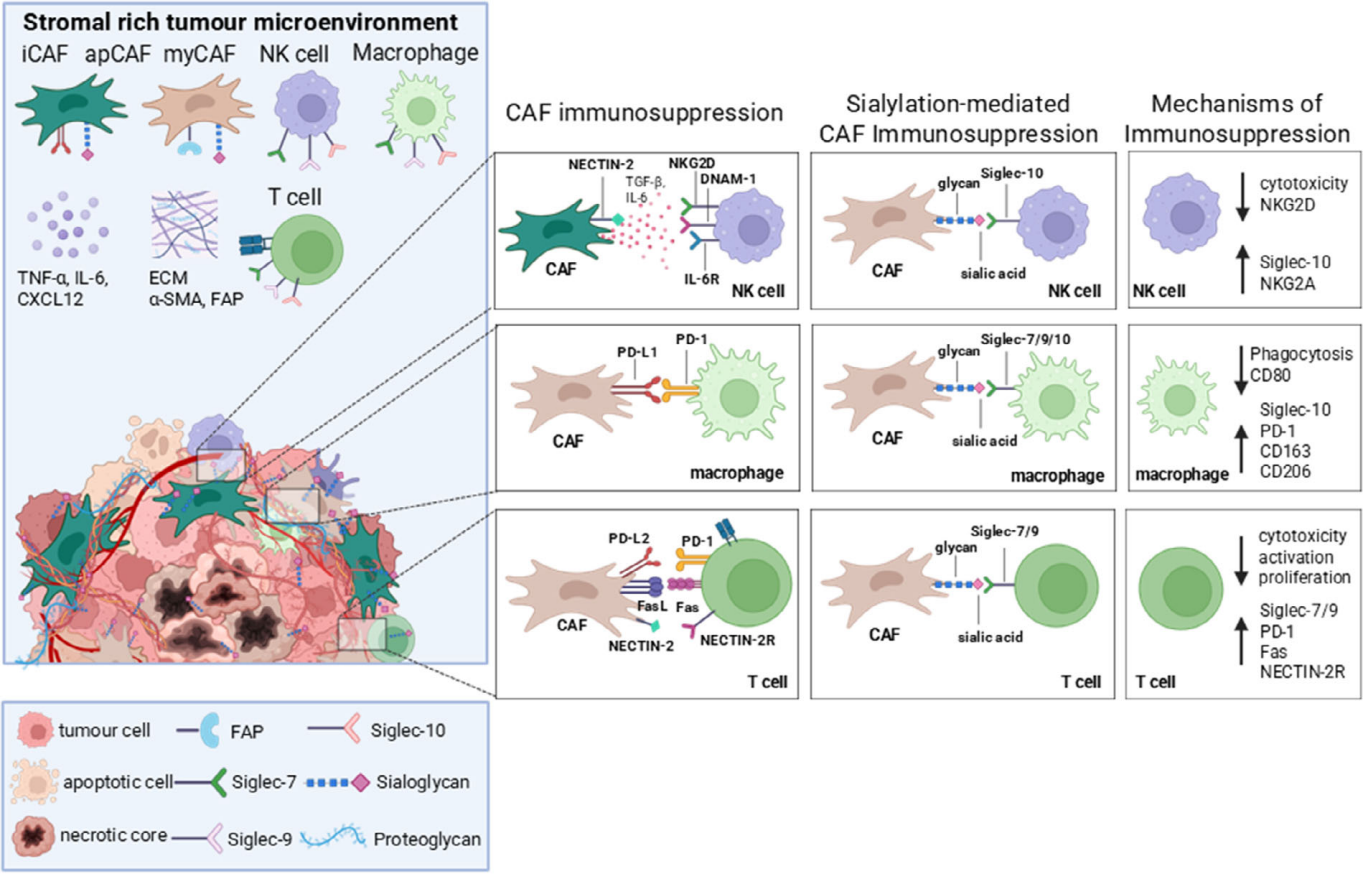
The stromal‐rich tumour microenvironment is associated with immunosuppression. Stromal cells in the TME, including both MSCs and CAFs, act as a protective barrier for tumour cells, surrounding the tumour epithelium, and can impact immune infiltration into the tumour. Multiple CAF subsets, including but not limited to myCAFs, apCAF and iCAFs can orchestrate immunosuppression by multiple mechanisms. iCAFs are associated with cytokine and chemokine secretion, such as TNF‐α and IL‐6, which inhibit immune cell activation, such as NK cells via NKG2D receptor engagement [105]. CAFs have been shown to express PD‐L1 and PD‐L2 that suppress T cell and macrophage functions [6, 7, 53]. myCAFs are associated with TGF‐β signalling and ECM components such as proteoglycans that contribute to mechanical stiffness in the TME, inhibiting drug and immune cell infiltration and are associated with resistance to immunotherapy. Breast cancer and CRC CAFs have been shown to express NECTIN2 [60], a DNAM‐1 ligand that can be sialylated [106] and can act as a decoy receptor for NK cells [105]. Evidence for sialylation‐mediated CAF immunosuppression includes elevated expression of surface sialoglycans and PD‐L1 expression in CRC [7, 53] and PDAC, resulting in modulation of T cell, NK cell and macrophage anti‐tumour functions [28, 29, 63]. Investigating the post‐translational modification of CAF markers, including sialic acid profiles of CAF markers, will be essential in developing CAF sialic acid‐targeting therapies.

The immunological hallmarks of stromal cells have been acknowledged and described in detail in recent years [4]. Stromal cells, including MSCs and CAFs, are orchestrators of immunosuppression in the TME (Figure 2) [59]. Multiple transcriptional subtypes of stromal cells and CAFs co‐exist in the TME, including inflammatory CAFs (iCAFs), antigen‐presenting CAFs (apCAFs) and myofibroblastic CAFs (myCAFs) and their characterisation has been recently reviewed [60, 61, 62]. Stromal cells in the TME express inhibitory ligands [6, 7, 53], secrete cytokines, chemokines and lipids that both induce apoptosis of T cells but also suppress innate and adaptive immune cells [4]. While tumour cell sialylation is well‐studied, recent evidence strongly suggests that sialylation on stromal cells, particularly CAFs, is a critical, and perhaps dominant, driver of immunosuppression in stromal‐rich cancers [28, 29, 63]. We showed for the first time that CRC CAFs modulate CD8+ T cell activity via the Siglec/sialic acid axis [29]. This was followed by observations by Boelaars et al. [28], who showed that hypersialylation of pancreatic ductal adenocarcinoma (PDAC) CAFs contribute to myeloid cell suppression, inducing TAM polarisation and suppressing anti‐tumour immune response. More recently, we showed that CRC‐derived CAFs express Siglec‐10 ligands that impact NK cell anti‐tumour function, which we propose is likely through engagement with Siglec‐10/G [63]. PDAC and CRC CAFs express higher levels of sialoglycans than cancer cells and can induce Siglec receptor expression on immune cells, suggesting that stromal cells can enhance Siglec receptor immunosuppressive signalling through enhanced ligand and receptor expression in the TME. A recent study from Jiang et al. [64] investigated dysregulated sialylation profiles in GC, highlighting that sialic acid metabolism is upregulated in the stromal‐rich subtype of GC. The identity of the stromal sialoglycans in these cancers, however, remains elusive. While several CAF markers such as fibroblast activation protein (FAP), CD105 and MUC1 have well‐characterised functional roles influenced by PTMs, the impact of glycosylation and sialylation on many other CAF markers remains underexplored. Addressing the knowledge gap in our understanding of the functional impact of glycan modification on CAFs in the TME will be critical to uncover novel therapeutic targets to modulate stromal‐immune interactions in cancer. These findings highlight stromal cell sialylation as an emerging, critical mechanism of tumour immune escape, of both the adaptive and innate immune compartments, with further mechanistic studies required to fully elucidate the therapeutic potential of targeting CAF glyco‐immune checkpoints.

Hypersialylation of β1 integrins mediated by ST6Gal1 has been shown to increase cell migration and invasion in in vitro models of the TME [65]. Interestingly, β1 integrin is considered a marker for a tumour‐specific CAF subtype, S4‐CAFs [66]. β1 integrins on S4‐CAFs may be hypersialylated, interacting with immune cell Siglec receptors and promoting CAF‐mediated immunosuppression in cancer. Investigating the β1 integrin sialylation profile of CAFs will be essential to confirm this mechanism. Additionally, sialylation of epidermal growth factor receptor (EGFR) by ST6Gal1 induces resistance to the EGFR‐targeting inhibitor gefitinib [42]. These findings strongly suggest a critical role for ST6Gal1 in the generation of hypersialylated receptors on CAFs that could modulate response to ligands and initiate pro‐tumorigenic signalling, contributing to cancer progression and therapeutic resistance. As fibroblasts have essential functions throughout the body, it will be imperative to target CAFs specifically to reduce off‐target effects in cancer treatment. Mechanistically, it may be important to understand the influence of hypersialylation on the functions of specific CAF glycoproteins and/or glycolipids. Table 1 summarises the sialylation profile of CAF markers and their association with different cancers.

**TABLE 1:** CAF biomarkers and potential for sialylation. These are characterised as established (strong experimental evidence of a functional impact of sialylation), early/preliminary evidence (indirectly linked to sialylation) and unknown (no evidence is available, or glycosylation status is unknown).

Marker	CAF subset	Cancer	Evidence for PTMs	Impact of PTMs	References
Fibroblast activation protein (FAP)	myCAFs, CAF‐S1, matrix‐remodelling CAFs, iCAFs	Stromal‐rich cancers including breast, colorectal, lung, pancreatic, ovarian	FAP expression by CAFs has been shown to be N‐linked glycosylated, contributing to tumour progression by remodelling ECM	Established	[33, 67, 68]
Wnt family member 2 protein (Wnt2)	iCAFs	OSCC and CRC	Modified by N‐linked glycosylation which affects interactions with cell surface receptors. WNT‐2 is overexpressed in cancers promoting tumour growth, angiogenesis and metastasis	Preliminary evidence	[31, 69, 70]
Leucine rich repeat containing 15 (LRRC15)	LRRC15+ CAFs	Sarcoma, glioblastoma, melanoma	LRRC15 is an extracellular glycoprotein mediating the interactions between stroma, ECM and tumour in the TME	Unknown	[71, 72, 73]
Cluster of differentiation 73 (CD73)	iCAFs, MSC‐CAFs	Hepatocellular carcinoma (HCC)	The adenosine‐producing function of CD73 is compromised in HCC due to aberrant N‐linked glycosylation	Established	[74, 75, 76]
Protein S100‐A4 (FSP1)	Migratory CAFs	Breast, colorectal, pancreatic, gastric	Involved in metastasis, inflammation and migration. The glycosylation status of S100 proteins influences stability and secretion	Preliminary evidence	[77, 78]
Platelet‐derived growth factor (PDGFRα/β)	PDGFRα+ CAFs, PDGFRβ+ CAFs	Glioblastoma (GBM)	ST6GAL1‐mediated sialylation of PDGFRβ increases glioblastoma growth	Established	[79, 80, 81]
Podoplanin (PDPN)	iCAFs	Squamous cell carcinoma, glioma, cervical cancer, pancreatic cancer	PDPN is a mucin‐type glycoprotein that plays a significant role in the TME such as immune interactions and EMT	Preliminary evidence	[82, 83, 84]
Vimentin	MSC‐CAFs	Breast, colorectal, lung, prostate, pancreatic	Aberrant sialylation of vimentin has been linked to enhanced invasiveness and metastasis	Preliminary evidence	[23, 85, 86]
Integrin β1 (CD29)	MSC‐CAFs/matrix remodelling‐CAFs	Breast cancer	Integrin β1 is α2,6 sialylated in MDA‐MB‐213 breast cancer cells	Preliminary evidence	[87, 88]
Mucin‐1 (MUC‐1)	Secretory CAFs	Colorectal, brain, metastatic breast cancer	MUC1 is a highly glycosylated, known Siglec 9 ligand	Established	[89, 90, 91]
CD43	myCAFs, iCAFs	AML	Sialylation of CD43 is specific to cancers demonstrating immunotherapeutic potential	Preliminary evidence	[92]
CD105	CD105+ CAFs	Breast, lung, colorectal, ovarian, HCC, pancreatic	Sialylation of CD105 plays a role in angiogenesis and tumour progression. CD105 is a co‐receptor for TGF‐β and is upregulated in many cancers	Established	[93, 94, 95]
Endo180	Matrix remodelling CAFs, myCAFs	Breast, colorectal, lung, head and neck, melanoma	ECM remodelling and invasion	Unknown	[5, 96]
Netrin G1 (NTNG1)	Neurogenic CAFs	PDAC, lung, colorectal	NetG1 is a glutamatergic pre‐synaptic protein. NetG1 expression correlates with poor prognosis in PDAC. NetG1+ CAFs are immunosuppressive and inhibit NK cell function	Unknown	[97]

## 6 Therapeutic Targeting of CAF Sialylation in Cancer

Therapeutic targeting of novel glyco‐immune checkpoints in the stromal‐rich TME has the potential to enhance an immunologically “hot” microenvironment, thereby improving anti‐cancer immunity and response to immunotherapies in immunosuppressive tumours.

**FIGURE 3:**
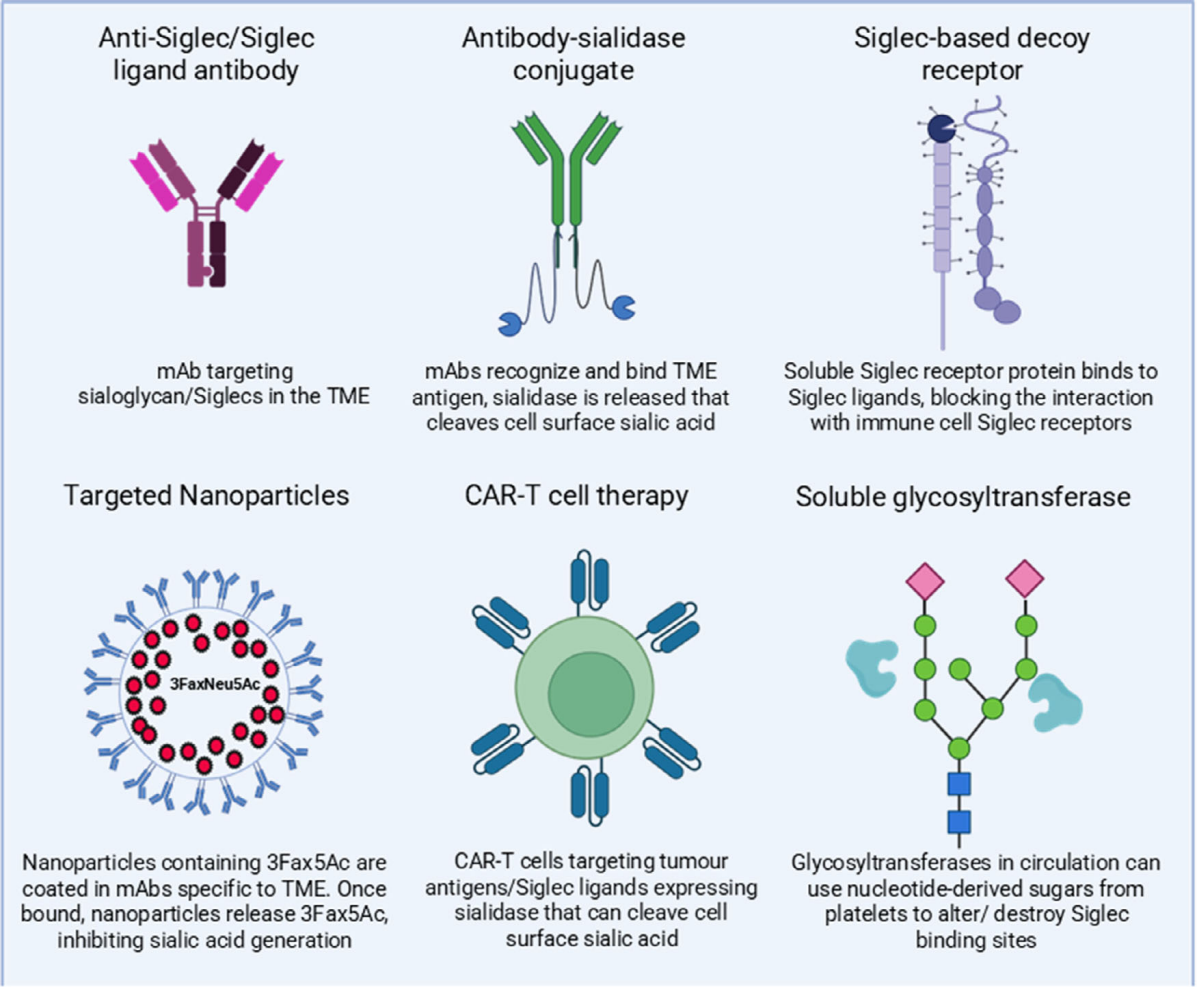
Approaches to target the sialic acid/Siglec axis in cancer. Novel targeting approaches for the Sialic acid/Siglec axis aim to target Siglecs and Siglec ligands as tumour antigens, but also as a target to reverse immunosuppression and reactivate anti‐tumour effector cells. These approaches include monoclonal antibodies (mAbs), antibody–drug conjugates (ADCs), small molecule inhibitors, decoy receptors (including Antibody‐lectin chimeras ‐Ablecs), nanoparticles and chimeric antigen receptor T cell (CAR‐T) therapy. Identification of specific Siglec ligands in various TMEs will enable more precise approaches. There have been multiple examples of the effectiveness of these therapy modalities in recent years. Examples include anti‐Siglec/Siglec ligand mAbs, [99] sialidase‐conjugated mAbs as ADCs [48, 100, 101], sialyltransferase inhibitor‐loaded nanoparticles [102], Ablecs [107] and sialidase‐conjugated CAR‐T cells [103]. Soluble Siglec decoy receptors, which bind to Siglec ligands, block the interaction of immune cell Siglec receptors with their Siglec ligands. Soluble glycosyltransferases could also be used therapeutically to use platelet‐derived nucleotide sugars to alter the glycocalyx in the TME.

In the TME, it is evident that the sialic acid/Siglec axis controls multiple anti‐tumour immune effector cells, including cytotoxic T cells, macrophages, dendritic cells and NK cells [16]. Given the crucial role of stromal sialylation in mediating immunosuppression, targeting this modification represents a promising strategy to enhance the function of many immune effector cells and promote anti‐tumour immunity, particularly in resistant, stromal‐rich cancers. Sialic acids can be targeted at multiple levels, including blocking Siglec receptor–ligand interactions, enzymatically removing sialic acids from glycans, or inhibiting their biosynthesis [98]. As Siglec‐sialic acid interactions function as immune checkpoints, disrupting these pathways has garnered attention in recent years (Figure 3). Although not yet approved clinically, the emerging area of glyco‐immune checkpoint inhibitors has some exciting, novel strategies making their way through pre‐clinical and clinical development. These strategies have already shown anti‐tumour effiacacy in multiple models and cancer types and therefore hold great promise for the field of cancer immunotherapeutics.

Anti‐Siglec ligand antibodies block the interaction of sialoglycans with Siglec receptors, inhibiting immune suppression. As well as direct targeting of Siglec receptors as tumour antigens [99], mAbs may be conjugated with sialidase to cleave sialic acids to activate anti‐tumour immune cells. In a breast cancer model, a HER2‐targeting mAb, trastuzumab, was tagged with sialidase, which increased cancer cell killing [100]. Sialic acid depletion, possibly through the use of sialyltransferase inhibitors or sialidases, has been shown to increase antibody–drug conjugate (ADC) delivery and killing of cancer cells [101]. ADCs have been successful in the clinic, with many FDA‐approved therapies for various cancers, including breast cancer and lymphoma [102]. Antibody‐conjugated nanoparticles (ACNPs), coated in monoclonal antibodies specific to a tumour or stromal antigen with a drug cargo that can be released upon binding, are emerging advances in ADCs. This is a highly specific technology, delivering the drug, including sialidase or sialyltransferase inhibitor, directly to the tumour site, negating the potential for off‐target toxicity. To overcome poor response to immunotherapies, researchers have devised combination strategies, incorporating high‐level immunotherapy engineering such as bispecific T cell engager (BiTE) therapy and, more recently, dual‐targeting engineered CAR‐T cell therapies [103]. Combining sialidase‐targeting of tumours with immunotherapy has been shown to increase therapeutic efficacy. Wu et al. [21] showed that desialylation of cancer cells enhanced CAR‐macrophage (CAR‐iMac) infiltration and subsequently prolonged survival in tumour‐bearing mice in vivo. Xiao et al. [27] recently showed that targeting the desmoplastic stroma of PDAC using a FAP‐CAR‐T cell approach increased cytotoxic T cell and NK cell infiltration, and enhanced response to PD‐1 immunotherapy. These studies highlight the therapeutic potential of novel targeting techniques to modulate the tumour and stromal compartments of the TME. Another exciting approach to targeting the Sialic Acid/Siglec axis are the development of antibody‐lectin chimeras (Ablecs). Ablecs are bispecific antibody‐like molecules with a cell‐targeting antibody domain and a lectin ‘decoy receptor’ domain that can target glycans and prevent signalling following enagagement with inhibitory receptors. Stark et al. recently demonstrated that Ablecs can potentiate macrophage phagocytosis and cytotoxicity and enhance their anti‐tumour effector mechanisms and can synergise with immune checkpoint blockade in multiple cancer models [107]. These approaches may be utilised to selectively identify stromal cells by targeting CAF/stromal‐specfic antigens, such as FAP, LRRC15 and others known CAF targets. Targeting stromal cell sialylation represents a novel and underexplored approach to overcoming immunosuppression in stromal‐rich tumours and has the potential to enhance immunotherapeutic efficacy in desmoplastic tumours. Novel therapeutic strategies that disrupt the physical stromal barrier and target the tumour glyco‐code offer a promising novel mechanism of tumour‐targeting in resistant cancers.

## 7 Summary and Future Perspectives

CAFs have been implicated in anti‐cancer therapeutic resistance and immunosuppression in stromal‐rich tumours for some time. Although this identifies CAFs as an important target in the TME, they are inherently heterogeneous, which presents challenges for the targeting of CAF‐specific markers in the TME. More recently, sialylation has emerged as a driver of immunosuppression, progression and invasion in cancer. Here, we suggest that CAF biomarkers may exhibit dysregulated sialylation patterns, dictates anti‐tumour immune responses through the Siglec receptor/ligand axis. Understanding the role of sialylation in CAF subsets, including iCAFs, myCAFs, and apCAFs, may be important in uncovering novel targets that promote immunosuppression. Sialylated proteins/lipids may also alter the function of these cells in both a Siglec‐dependent and independent manner and further research is needed to define these functions. We and others recently reported that stromal cells exhibit higher sialylation than cancer cells and modulate anti‐tumour immune responses via Siglec receptor engagement. Hypersialylation has been strongly associated with immune evasion in cancer, where sialylated tumour cells evade immune clearance. Recent evidence suggests that glycosylation of immune checkpoints in the TME may alter their function. De‐glycosylation and desialylation approaches hold promise for reactivation of immune cells in the TME through the disruption of ICI interactions with their receptors, opening a new avenue for ICI targeting. It is not fully understood whether sialylation of ICIs regulates their function in the TME, and this knowledge may open up existing therapeutic possibilities in the future. Identification of CAF subset‐specific markers, as well as their sialylation profile, and identification of mechanisms of immunosuppression, will undoubtedly aid in the development of sialic acid‐targeting therapies. This approach may hold the potential to reverse immunosuppression and improve response to immunotherapies in stromal‐rich, therapy‐resistant cancers.

## Author Contributions

Aoise O'Neill was involved in the idea concept and design, figure preparation, literature search and data acquisition, interpretation of data, critical evaluation of data, writing and revision and final approval of manuscript. Norashikin Zakaria, Hannah Egan, Oliver Treacy, Aisling M. Hogan, Michael O'Dwyer, and Sean O. Hynes were involved in concept and design, interpretation of data, critical evaluation of data, reviewing and finalisation of the manuscript. Aideen E. Ryan was involved in idea, concept and design, figure preparation, literature search and data acquisition, interpretation and critical evaluation of data, writing and revision and final approval of manuscript. All authors have approved the final version of the manuscript.

## Ethics Statement

This manuscript does not report any experiments that were conducted with animals and does not contain human studies.

## Conflicts of Interest

M O'D and A.E.R. are co‐inventors on related patent US20210186999A1. The remaining authors declare no conflicts of interest.

## Data Availability

The authors have nothing to report.
